# The role of E3 ubiquitin ligases in the development and progression of glioblastoma

**DOI:** 10.1038/s41418-020-00696-6

**Published:** 2021-01-11

**Authors:** Luke M. Humphreys, Paul Smith, Zhuoyao Chen, Shahd Fouad, Vincenzo D’Angiolella

**Affiliations:** Department of Oncology, Medical Research Council Institute for Radiation Oncology, University of Oxford, Oxford, UK

**Keywords:** Ubiquitin ligases, CNS cancer

## Abstract

Despite recent advances in our understanding of the disease, glioblastoma (GB) continues to have limited treatment options and carries a dismal prognosis for patients. Efforts to stratify this heterogeneous malignancy using molecular classifiers identified frequent alterations in targetable proteins belonging to several pathways including the receptor tyrosine kinase (RTK) and mitogen-activated protein kinase (MAPK) signalling pathways. However, these findings have failed to improve clinical outcomes for patients. In almost all cases, GB becomes refractory to standard-of-care therapy, and recent evidence suggests that disease recurrence may be associated with a subpopulation of cells known as glioma stem cells (GSCs). Therefore, there remains a significant unmet need for novel therapeutic strategies. E3 ubiquitin ligases are a family of >700 proteins that conjugate ubiquitin to target proteins, resulting in an array of cellular responses, including DNA repair, pro-survival signalling and protein degradation. Ubiquitin modifications on target proteins are diverse, ranging from mono-ubiquitination through to the formation of polyubiquitin chains and mixed chains. The specificity in substrate tagging and chain elongation is dictated by E3 ubiquitin ligases, which have essential regulatory roles in multiple aspects of brain cancer pathogenesis. In this review, we begin by briefly summarising the histological and molecular classification of GB. We comprehensively describe the roles of E3 ubiquitin ligases in RTK and MAPK, as well as other, commonly altered, oncogenic and tumour suppressive signalling pathways in GB. We also describe the role of E3 ligases in maintaining glioma stem cell populations and their function in promoting resistance to ionizing radiation (IR) and chemotherapy. Finally, we consider how our knowledge of E3 ligase biology may be used for future therapeutic interventions in GB, including the use of blood–brain barrier permeable proteolysis targeting chimeras (PROTACs).

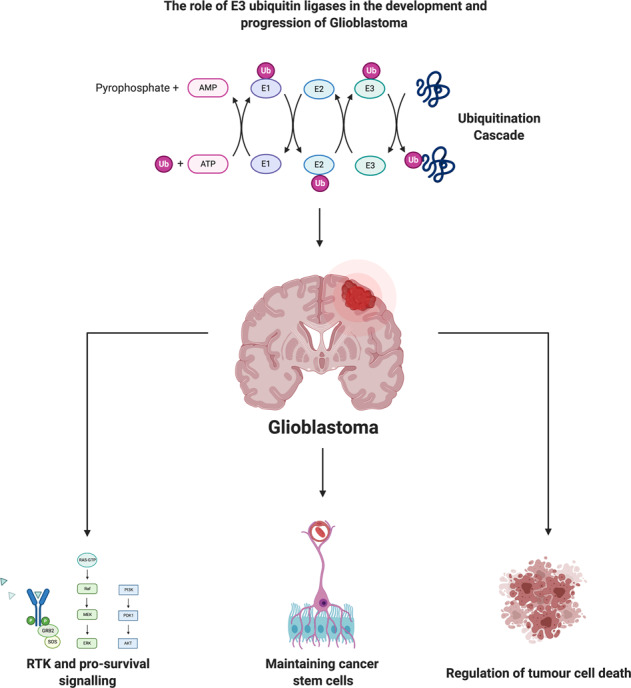

## Facts

- GB is an aggressive form of brain cancer which presents with a poor prognosis and has limited treatment options.
- In GB, E3 ubiquitin ligases regulate important cellular functions, such as RTK pro-survival signalling and DNA damage repair.
- E3 ubiquitin ligases play multifaceted roles in the maintenance of GSCs, a population of cells thought to be responsible for GB disease recurrence.
- E3 ubiquitin ligases alter the sensitivity of GB cells to standard-of-care therapies such as IR and DNA alkylating agents.

## Open questions

- What role do other E3 ubiquitin ligases (not discussed here) play in lineage specification of neural stem cells?
- What are the potential E3 ligases to inhibit in GB to improve treatment?
- Can E3 ligases be targeted to promote the differentiation of GSCs?
- Can we exploit PROTACs to drive differentiation of GSCs?
- Can we identify E3s with cancer-specific functions that synergise in killing tumour cells with IR and temozolomide?
- Can we exploit genetic losses common in GB via synthetic lethal approaches?
- How do ubiquitin chain topologies impact GB disease progression?

## Introduction

Glioma is an umbrella term for primary brain tumours which are classified according to their cell of origin, and include astrocytic tumours, ependymomas and oligodendrogliomas. Glioblastoma (GB) (WHO Grade IV astrocytoma) is a heterogeneous disease that can occur de novo or as secondary disease. De novo cases account for ~90% of all GB and arise spontaneously, whereas secondary GB has a classical, progressive natural history, and develop from low-grade astrocytic tumours. Histologically, primary and secondary tumours share similar phenotypes and are therefore not easily distinguishable (see Supplementary Text 1—Histology of GB). On a molecular level, secondary GB is characterised by a mutation in the metabolic enzyme isocitrate dehydrogenase 1, which occurs in >80% of secondary GB and <5% of primary GB cases [1–3]. Importantly, secondary GBs have a significantly better prognosis than primary GB cases [3]. The molecular classification of GB is discussed in detail in Supplementary Text 2—Molecular Classification of GB.

Current treatment options for GB are limited. Following diagnosis, patients usually undergo surgical resection, followed by concomitant radiotherapy and chemotherapy. The recommended first-line chemotherapeutic is the DNA alkylating agent temozolomide (TMZ; Temodar) [4], an orally administered prodrug, which is converted to 5-amino-imidazole-4-carboxamide and methylhydrazine in cells. It is cytotoxic due to its ability to add methyl groups to DNA (N^7^ and O^6^ on guanines and O^3^ on adenines), leading to errors in DNA replication and subsequent cell death [5]. O^6^ alkylation is the most toxic alteration induced by TMZ and can be reversed by O-6-methylguanine-DNA methyltransferase (*MGMT*) [6], the expression of which leads to treatment failure [7]. Methylation of the *MGMT* promoter is used to predict prognosis regarding TMZ treatment. Clinical trials demonstrated that patients with methylated *MGMT* treated with TMZ had a median survival of 21.7 months compared with 15.3 months for radiotherapy alone. This survival benefit was lost in patients lacking *MGMT* promoter methylation [7].

Despite significant advances in our understanding of GB, 5-year survival remains at a dismal 5% [8]. TMZ remains one of the only chemotherapeutic options, indicating a dire need for alternative treatments. In this review, we focus on the role of E3 ligases in GB development and progression and the potential of targeting this class of enzymes.

## E3 ubiquitin ligases

**Fig. 1:**
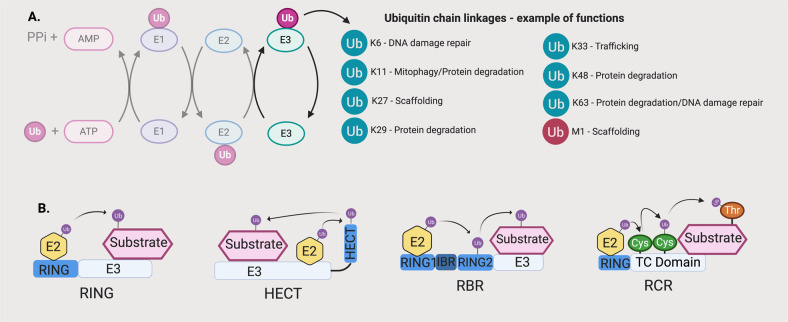
The ubiquitin system and families of E3 ubiquitin ligases. **a** Ubiquitin is ligated to proteins via a cascade involving E1, E2 and E3 ubiquitin ligases. E3s attach ubiquitin via specific lysine residues, which determine protein fate following ubiquitin conjugation. Ligation is carried out by a family of four distinct E3 ligases (**b**).

Ubiquitylation is a reversible, post-translational modification that is regulated by a large family of proteins known as E3 ubiquitin ligases. E3s target a broad spectrum of substrates involved in a myriad of cellular processes including metabolism, DNA repair and programmed cell death. Aberrant functions of E3s are linked to many human diseases, hence E3s represent an important class of drug targets. An overview of E3 ubiquitin ligases’ mechanism of action and classification is provided in Fig. 1 and Supplementary Text 3—Overview of E3 Ubiquitin Ligases.

## E3 ubiquitin ligases in GB: receptor tyrosine kinase (RTK) signalling

### RTK signalling receptors

**Fig. 2:**
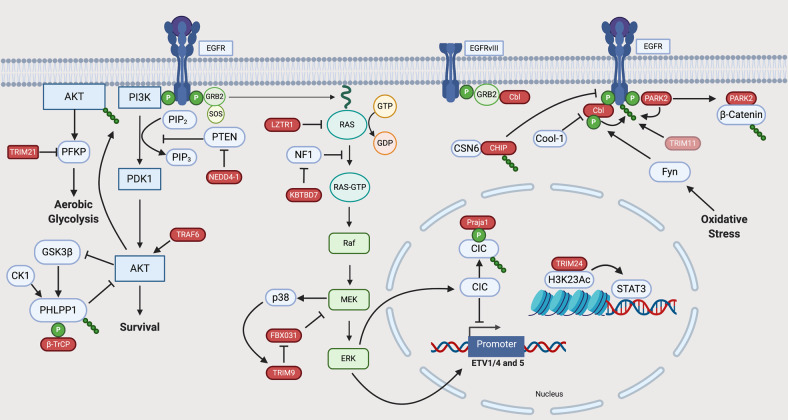
The role of E3 ubiquitin ligases in RTK signalling in GB. RTKs are transmembrane receptors containing extracellular, transmembrane and intracellular portions. The extracellular domain interfaces with the extracellular milieu allowing expressing cells to react and adapt to extracellular signals. Upon binding their cognate ligands, RTKs undergo autophosphorylation leading to downstream signalling [156]. In this way, RTKs transduce extracellular cues as signals into a cell. EGF signalling is a prototypical example of altered RTK signalling in GB. Activation of the receptor leads to phosphorylation of c-terminal residue tyrosines, facilitating the docking of Src homology (SH) domain-containing proteins and activation of several pathways, including the MAPK pathway, signal transducers and activators of transcription (STAT) signalling and Src-dependent phosphoinositide 3-kinase (PI3K)/Akt signalling [157]. At the membrane, E3s such as c-CBL can decrease oncogenic signalling by promoting EGFR ubiquitylation and turnover. RTK signalling converges on several pathways including the phosphoinositide 3-kinase (PI3K)/Akt pathway. Activation-dependent phosphorylation of RTKs (such as EGFR) facilitates the binding of class 1a PI3K via SH2 domains. This leads to the activation of PI3K and subsequent phosphorylation of phosphatidylinositol 4,5-bisphosphate to generate phosphatidylinositol 4,5-triphosphate (PIP_3_) [158]. Via pleckstrin homology (PH) domains, Akt binds PIP_3_ where it is phosphorylated by phosphoinositide-dependent protein kinase-1 (PDK-1). An important negative regulator of this pathway is the phosphatase and tensin homologue (PTEN), a protein whose gene is mutated/deleted in 41% of GB cases. Akt signalling can also be modulated via negative feedback loops with phosphatases including PH domain leucine-rich repeat protein phosphatase (PHLPP) [159, 160]. The MAPK pathway is an important downstream signalling pathway activated by RTKs. As  one of the most commonly mutated pathways in human cancer, it translates  extracellular signals into cellular phenotypes such as proliferation, differentiation, migration and invasion [161]. Following RTK activation, son of sevenless (Sos) is recruited to the plasma membrane via interaction with Grb2. Sos is a guanine nucleotide exchange factor (GEF) which promotes the activation of membrane-bound RAS via binding of guanosine triphosphate (GTP) with Ras. Active Ras can then promote multiple oncogenic cellular responses. In contrast, GTPase-activating proteins (GAPs) accelerate Ras-mediated GTP hydrolysis and act as negative regulators of Ras signalling and are therefore tumour suppressors [162]. Red icons represent E3 ligases and their functions as observed in GB models. Where specific substrates are yet to be identified, E3 ligases appear as pink icons.

In GB, 67% of cases have alterations in at least one RTK [9]. The most frequently altered RTK is EGFR. The roles of E3 ligases in mediating EGFR signalling in GB are summarised in Fig. 2 and Table 1 and described in detail below.

**Table 1:** Modulation of receptor tyrosine kinase signalling by E3 ligases.

E3 ligase	Substrate/Downstream effector	Pathway	Reference(s)
CBL	EGFR	EGFR signalling	[10, 13–15, 19–22, 25]
CHIP	EGFR	EGFR signalling	[31, 32]
KBTBD7	NF1	MAPK	[64]
LZTR1	RAS	MAPK	[51–54]
NEDD4-1	PTEN	PI3K-AKT	[44, 45, 56]
PARK2	EGFR, PKM2	EGFR signalling/PI3K-AKT	[27, 28, 42]
PRAJA1	CIC	EGFR signalling MAPK	[163]
SCF^βTrCP^	PHLPP1	PI3K-AKT	[35, 37]
SCF^FBXO31^	MKK6	MAPK	[67]
SCF^SKP2^	P27^Kip1^	Cell cycle	[47, 48]
TRAF6	Akt	PI3K-AKT	[39, 40]
TRIM9_(S)_	MKK6	MAPK	[68]
TRIM11	–	EGFR signalling	[29]
TRIM21	PFKP	PI3K-AKT	[43]

Casitas B-lineage lymphoma (Cbl) is a well-characterised E3 ligase involved in regulating EGFR signalling and other RTKs through receptor recycling at the membrane [10–12]. Following receptor activation, Cbl binds directly to p-Y1045 on the c-terminus of EGFR or via Grb2, leading to phosphorylation of Cbl on Y371, a critical requirement for Cbl E3 ligase activity [13]. Cbl-dependent ubiquitination of EGFR leads to clathrin-mediated internalisation of the receptor and sorting into lysosomes where the receptor is degraded [10, 14, 15], leading to reduced EGFR signalling. In GB, ~20% of classical tumours express a truncated form of EGFR that lacks exons 2–7 (EGFRvIII) [9, 16] and is constitutively active. The signalling activity of EGFRvIII is lower than wild-type EGFR and Y1045, the critical docking residue for Cbl, is hypophosphorylated [17, 18]. Whilst there are conflicting studies [19], Cbl is thought to interact primarily with EGFRvIII through Grb2, which attenuates Cbl-dependent internalisation and degradation of the EGFRvIII receptor leading to sustained proliferative signalling [20]. The Cbl-EGFR axis undergoes additional levels of regulation. For example, EphrinA5 expression enhances Cbl binding and ubiquitination of the EGFR receptor, which increases the internalisation and degradation of the receptor in GB models [21]. Further, a novel pathway regulating Cbl activity, termed the Redox/Fyn/Cbl pathway, was discovered in oligodendrocyte-type-2 astrocyte [O-2A] progenitor cells/oligodendrocyte precursor cells (O-2A/OPCs) whereby oxidative stress activates Fyn kinase leading to activation of Cbl and a subsequent reduction in RTK signalling in O-2A/OPCs [22]. In GB models, induction of oxidative stress by bis-chloroethylnitrosourea (BCNU/carmustine), an alkylating agent commonly used in the treatment of glioma, did not lead to c-Cbl phosphorylation or a decrease in EGFR activity. It was shown that Cool-1, a protein involved in the activation of the Ras-like family of Rho proteins, prevented Cbl activity, maintained stemness and the ability of GB cells to initiate tumours in vivo in a pathway that is distinct from that seen in other tumours [23–25]. In addition, tissue transglutaminase, which has upregulated transcript levels in 45% of mesenchymal and 16% of classical GB, can bind to and inhibit Cbl activity, resulting in stabilisation of EGFR and sustained oncogenic signalling [26].

PARK2 is an E3 ubiquitin ligase that is frequently either lost (on chromosome 6q) or mutated in GB [27]. It can modulate EGFR expression at the protein level via direct binding and ubiquitination and at the mRNA level via indirect transcriptional regulation [28]. PARK2 can also act as a tumour suppressor via ubiquitination-dependent degradation of β-catenin, leading to attenuation of Wnt signalling [28]. Tripartite motif-containing protein (TRIM) 11 promotes increased EGFR levels in glioma. In addition to being overexpressed in gliomas, knockdown of TRIM11 in primary glioma cultures was sufficient to significantly reduce levels of EGFR [29]. Finally, carboxyl terminus of heat-shock protein 70-interacting protein (CHIP), a U-box E3 ligase, can also regulate EGFR expression in GB. CHIP is destabilised by the COP9 signalosome (CSN), a multi-subunit protein complex which is known to regulate CRL family E3 ligases via its de-neddylation activity [30]. Interactions between CSN6, a subunit of the CSN, and CHIP, promote CHIP’s autoubiquitination and degradation. Degradation of CHIP leads to stabilisation of EGFR, facilitating oncogenic signalling [31, 32].

### RTK signal transduction—PI3K-AKT

The PI3K/Akt pathway is altered in ~90% of GB cases [9]. The roles of E3 ligases in mediating PI3K/Akt signal transduction in GB are described in detail below and summarised in Fig. 2.

Pleckstrin homology domain leucine-rich repeat protein phosphatase (PHLPP1) negatively regulates Akt signalling via the removal of a phosphorylation in the hydrophobic motif of Akt [33]. Following phosphorylation by casein kinase I (CK1) and glycogen synthase kinase 3β (GSK3β), PHLPP1 is recognised by the substrate-binding F-box protein β-TrCP. β-TrCP is a substrate recognition protein of the Skp-Cullin 1-F-box protein complex (SCF^β-TrCP^) which recognises the degron sequence equal or similar to DSGXXS in which both serines are phosphorylated [34]. Recognition of PHLPP1 by SCF^β-TrCP^ leads to its ubiquitination and degradation via the proteasome [35, 36]. Akt-mediated phosphorylation and inactivation of GSK3β stabilises PHLPP1, generating a negative feedback loop [35]. In astrocytoma cell lines, β-TrCP is located in the cytoplasm and therefore can interact with phosphorylated PHLPP1. However, in GB cell lines and primary glioma neurospheres, β-TrCP-1 is localised within the nucleus, where it can no longer interact with PHLPP1. Paradoxically, this shift in the spatial distribution of β-TrCP-1 does not alter the stability of the PHLPP1 protein but make PHLPP1 levels insensitive to PI3K inhibitors, indicating a non-functional feedback loop [37]. SCF^β-TrCP^ has many other substrates and the human genome codes for two paralogs (namely β-TrCP-1 and β-TrCP-2 or FBXW1 and FBXW11), therefore it is difficult to reconcile these findings with the fact that alterations of β-TrCP would result in defective degradation of many other substrates. Alteration of β-TrCP-1 and β-TrCP-2 localization could have much larger effects on cell survival.

Ubiquitylation enhances the translocation and activation of Akt on the cell membrane [38]. The E3 ligase responsible for this ubiquitylation differs according to the growth factor receptor involved. For example, tumour necrosis factor receptor-associated factor 6 (TRAF6) is implicated in IGF-1-mediated ubiquitylation of Akt and the SCF^Skp2^ complex is important for EGF-dependent Akt ubiquitylation in breast cancer cell lines and mouse embryonic fibroblasts (MEFs) [38, 39]. In GB, EGFR signalling leads to phosphorylation of discoidin, CUB and LCCL domain-containing protein 2 (DCBLD2) at Y750, which resides within a recognition sequence for TRAF6. Once phosphorylated, this single-pass membrane protein binds to TRAF6, recruiting it to the cell surface. This enhances the E3 ligase activity of TRAF6 and subsequent ubiquitylation of Akt increases proliferation in primary GB cell lines, GSCs and established GB cell line models [40].

Active Akt drives aerobic glycolysis in cancer cells in a process known as the Warburg effect [41]. PARK2 may play an important role in this metabolic switch as it can bind to and ubiquitylate pyruvate kinase M2 (PKM2), the predominant isoform of pyruvate kinase expressed in GB. PARK2-mediated ubiquitylation leads to lower PKM2 enzymatic activity and therefore a lower rate of glycolysis [42]. In addition to PKM2, phosphofructokinase-1 platelet (PFKP) isoform is a driver of the increased glycolytic rate observed in GB. PFKP stability can be regulated by the E3 ligase TRIM21, which ubiquitylates PFKP on its K10 residue resulting in proteasomal degradation. Akt-dependent phosphorylation of PFKP on Ser386 abrogates TRIM21-dependent PFKP ubiquitylation, thereby enhancing PFKP and pyruvate kinase activity and lactate production. PFKP loss in a U87/EGFRvIII in vivo model of GB significantly reduced tumour growth, highlighting the potential physiological importance of TRIM21 in GB growth [43].

PTEN negatively regulates Akt by converting PIP_3_ into PIP_2_ (Fig. 2). Whilst mutations and deletions of PTEN are common in GB, cells also exploit other mechanisms to deregulate the PTEN axis. NEDD4-1 was identified as an E3 ligase for PTEN, targeting it for degradation [44]. In GB, the transcription factor forkhead box protein M1B (FOXM1B) is overexpressed and can bind to the promoter of NEDD4-1. Overexpression of FOXM1B increases levels of NEDD4-1, which leads to downregulation of PTEN [45, 46]. In this way, the FOXM1–NEDD4-1 axis can regulate Akt signalling in the absence of PTEN deletion or mutation. PTEN can also regulate the ubiquitin-dependent degradation of the CDK inhibitor p27^KIP1^. Following phosphorylation of p27^KIP1^ on Thr187, it is recognised by the SCF^Skp2^ complex and ubiquitylated, leading to its degradation [47]. In GB, Skp2 functions as an important component of the PTEN/PI3-kinase pathway. Increasing PI3K activity leads to increased levels of Skp2 and subsequent degradation of p27, facilitating cell cycle progression [48]. Therefore, several regulatory processes modulated by E3 ubiquitin ligases converge on the PI3K/Akt pathway to regulate GB cell survival.

### RTK signal transduction—MAPK pathway

The MAPK pathway (Fig. 2) is frequently dysregulated in cancer. However, proteins such as Ras and BRAF are infrequently mutated in GB (1% and 2%, respectively) suggesting that negative regulators of the MAPK pathway may play an important role in GB development and progression [9].

Leucine zipper-like transcriptional regulator 1 (LZTR1) is a kelch domain-containing protein that acts as a substrate recognition protein in a Cullin-3 (Cul3) E3 ubiquitin ligase complex. Mutations in *LZTR1* have been reported in diseases such as schwannomatosis [49] and Noonan syndrome [50] whereby loss of LZTR1 drives de-differentiation and proliferation of cells. *LZTR1* is mutated or deleted in ~4% and ~ 20% of GB cases, respectively [9, 51]. Recently, a study using *LZTR1* hemizygous deleted MEFs identified Ras as a target of the Cul3–LZTR1 ubiquitin ligase complex. Ras was ubiquitylated on K170 preventing its hypervariable region from associating with the cell membrane and reducing its activation. LZTR1 also caused the relocalisation of Ras to endomembranes [52]. In genetic screens, inactivation of LZTR1 led to increased resistance to tyrosine kinase inhibitors as a result of enhanced MAPK signalling [53]. This study also demonstrated that GB-relevant mutations in *LZTR1* are loss-of-function mutations that increase Ras-dependent proliferation of cells [53]. Whilst these studies concluded that the effect of Cul3–LZTR1 on Ras was independent of degradation [52, 53], LZTR1 can degrade Ras in GB models [54]. *LZTR1* mutations identified in GB cluster on critical substrate recognition residues within the kelch domain and GB tumours that bear *LZTR1* mutations have a gene expression profile enriched for genes previously associated with glioma growth and neurosphere formation [51, 55]. Further, overexpression of LZTR1 is sufficient to prevent neurosphere formation and reduce the expression of GSC markers [51]. Taken together, these findings demonstrate that LZTR1 could be an important modulator of MAPK signalling, GB progression and GSC maintenance.

Hemizygous loss of *LZTR1* also led to reduced ubiquitylation on two other E3 ligases, NEDD4 and MycBP2 [52]. However, the importance and function of these ubiquitylation events remain unresolved. NEDD4 potentially regulates the PI3K pathway via its controversial ability to degrade PTEN [44, 56]. MycBp2 is a non-conventional E3 ligase that has esterification activity and selectively ubiquitylates threonine/serine residues and, potentially, non-protein substrates [57]. MycBp2 also forms a non-canonical SCF complex consisting of Skp1/FBXO45/MycBp2 (lacking Cul1) that controls neural development [58, 59]. It is also altered in GB [60] and can bind directly to MYC protein [61], which is frequently amplified in G-CIMP^+^ tumours [9]. The potential oncogenic role these E3 ligases play in the absence of LZTR1 has not yet been identified and the E3 ligases may warrant further investigation as potential therapeutic targets in this subset of tumours.

Neurofibromin (NF1) is a GTPase-activating protein that converts active GTP bound Ras to the inactive GDP bound form. It is mutated or deleted in ~10% of GB [9] likely resulting in prolonged Ras activation and tumour development [9, 62]. NF1 levels can be regulated by protein kinase C via Cul3-mediated ubiquitylation and proteasomal degradation [63]. Knockdown of Cul3 was sufficient to suppress Ras signalling and mass spectrometry revealed KBTBD7 as the substrate recognition protein responsible for NF1 ubiquitylation [64]. Suppressing Cul3 expression was also sufficient to reduce the growth of GB model tumours in vivo, whereas *NF1* null cells were unaffected by loss of Cul3 [64]. The Cul3–KBTBD7 E3 ligase complex can, therefore, modulate NF1 levels and indirectly regulate MAPK pathway activity. Of note, both LZTR1 and KBTBD7 use Cul3 as a core scaffold, thus, the respective contribution of these adaptors in Cul3 depletion experiments needs to be assessed.

Another member of the MAPK pathway, p38, is involved in a cell’s response to genotoxic stress and inflammation. In the brain, it regulates apoptosis and alters the tumour initiating capacity of glioma cells [65, 66]. Activation of p38 is dependent on the phosphorylation of its ‘TGY’ motif, which is a target of the map kinase kinase 6 (MKK6) enzyme. In GB models, MKK6 is regulated by both K63- and K48-linked ubiquitylation [67, 68]. The SCF^FBX031^ complex ubiquitylates MKK6 via K48 linkages leading to its degradation via the proteasome [67]. Conversely, the TRIM9_(s)_ E3 ligase can attach K63-linked ubiquitin onto K82 of MKK6 preventing SCF^FBX031^-dependent polyubiquitylation. Through a positive feedback loop, MKK6 phosphorylates and activates p38, which then phosphorylates TRIM9_(S)_ on Ser76/80 leading to its stabilisation, augmentation of p38 signalling and suppression of GB progression [68]. The balance between SCF^FBX031^ and TRIM9_(S)_ activity is, therefore, important for mediating the progression of GB.

Whilst not covered extensively in the main text, it is important to note that E3 ligases can also alter RTK signalling via interactions in the nucleus (Supplementary Text 4—RTK signalling – Nucleus and Table 1). In addition, they can influence the development and progression of GB via non-RTK based signalling pathways (Supplementary Text 5—Cancer signalling pathways beyond RTKs and Supplementary Fig. 1).

## E3 ligases and their role in maintaining GSCs

**Fig. 3:**
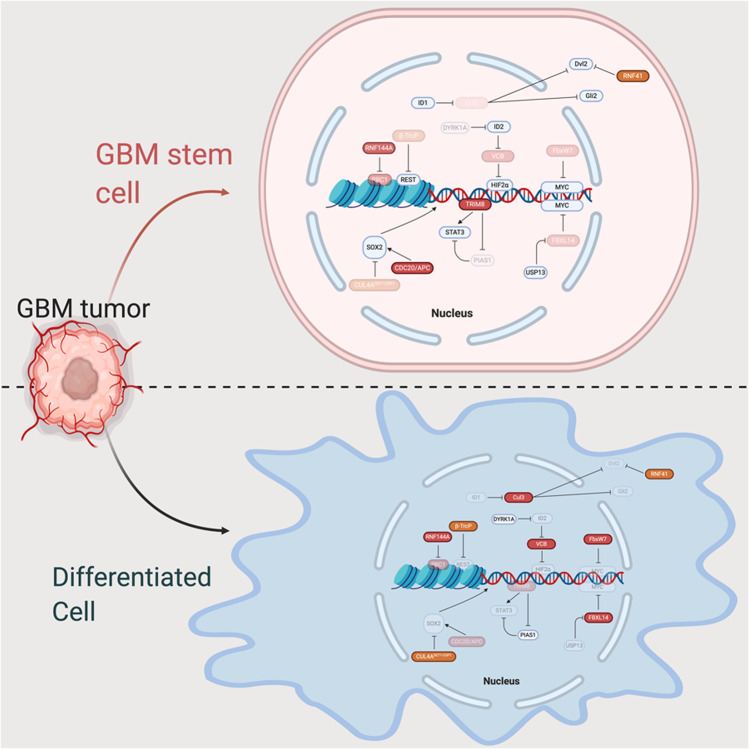
The role of E3 ubiquitin ligases in maintaining GSCs. GSCs are a population of de-deferentiated cells responsible for treatment failure and GB propagation. E3 ligases directly affect transcriptional programmes that mediate the differentiation of GSCs through direct interaction with or alteration of the stability of transcription factors involved in the stem cell transcriptional programme. Red icons represent E3 ligase functions shown directly in GSC/differentiated cell models. Pink icons represent reduced E3 ligase expression/activity. Orange coloured icons represent E3 ligase functions identified in neural stem cell or GB models.

Overall, GSCs exhibit higher ubiquitylation levels than their differentiated progeny and are hypersensitive to protease inhibitors, suggesting a critical role for the ubiquitin proteasome system in these cells [69]. The biology of GSCs is expertly reviewed elsewhere [70, 71] and is beyond the scope of this review. In this section, we review the role of E3 ligases in maintaining GSC populations. The details discussed in this section are summarised in Fig. 3 and Table 2.

**Table 2:** E3 ligase-dependent modulation of glioblastoma stem cells.

E3 ligase	Substrate/Downstream effector	Pathways	Reference(s)
BMI1	Histone 2A (lysine 119)	–	[73–75, 77, 78, 82–84, 86]
CDC20/APC	SOX2 stabilisation	Stem cell maintenance	[104]
Cul3	Dvl2, GLI2	SHH/Wnt	[89]
HUWE1	N-Myc	–	[99]
RNF19A	–	–	[106]
RNF144A	BMI1	–	[77, 78]
RNF41	Dvl2	SHH	[90]
SCF^βTrCP^	REST	Neural stem cell maintenance	[92–94, 96]
SCF^FBXL14^	Myc	–	[97]
TRIM8	PIAS1	STAT signalling	[101, 102]

BMI1 (RING finger protein 51 [RNF51]/Polycomb Group RING Finger Protein 4 [PCGF4]) is part of the polycomb repressor complex 1 (PRC1), an E3 ubiquitin ligase complex that monoubiquitylates histone 2A on K119 leading to chromatin compaction and repression of gene expression [72, 73]. BMI1 complexes with RING1A/B and the E2 UbcH5c to form the active ligase which has critical roles in maintaining the stem cell phenotype of GSCs [74, 75]. Another chromatin modifier, PRC2, regulates chromatin compaction via H3K27 trimethylation [76]. The stability of PRC1/2 components differs depending on the GSC subtype, altering their response to cellular stressors [77]. For example, in proneural perivascular GSCs, BMI1 is ubiquitylated by RNF144A (a member of the RBR family of E3 ligases) leading to its degradation. BMI1 degradation enhances the sensitivity of these cells to stressors such as hypoxia and increases their reliance on the PRC2 complex for survival. Conversely, in hypoxic mesenchymal GSCs, BMI1 is stabilised due to the reduced expression of RNF144A. This increases the resistance of these cells to stressors such as hypoxia and nutrient deprivation [77]. In addition, mesenchymal-specific expression of Zinc Finger DHHC-Type Palmitoyltransferase 18 (ZDHHC18) prevents BMI1 degradation via its ability to interact with RNF144A, inhibiting the BMI1–RNF144A interaction [78]. Importantly, this interplay between RNF144A and BMI1 renders mesenchymal GSCs sensitive to BMI1 inhibition and proneural GSCs sensitive to PRC2 inhibition. Both of these GSC types exist within GB, suggesting that dual targeting of these cells may be a promising therapeutic strategy [77].

BMI1 was first described as an oncogene that inhibited the expression of the Ink4a/Arf locus, which is critical for cell cycle regulation and whose expression is commonly attenuated in GB [9, 79–81]. It is expressed in many brain cells, particularly in the subventricular zone where neural stem cells (NSCs), considered a possible cell of origin for GB, and neural progenitors reside. Using Ink4a/Arf locus/BMI1 deficient NSCs stably expressing EGFRvIII, BMI1 was demonstrated to be important for the growth of brain tumours and overall survival following stereotactic injection of the cells into the brain of mice. In addition, deficiency in Ink4a/Arf and BMI1 led to decreased proliferation in EGFRvIII mutant astrocytes and generated lower-grade gliomas in vivo [82]. BMI1 is also important in GSCs’ response to TGFβ and endoplasmic reticulum stress signalling [83]. Moreover, shRNA of BMI1 in GSCs demonstrated that it suppresses multiple tumour suppressor pathways, including the major histocompatibility complex (MHC) cluster and Notch signalling, and is critical in maintaining stem cell renewal [84]. Furthermore, a frequently downregulated microRNA in GB, miR-128, is capable of targeting the 3′-untranslated region of BMI and when overexpressed can prevent GSC renewal [85]. Following irradiation of GSCs, BMI1 can recruit proteins involved in the DNA double-strand break response and non-homologous end joining. Knockdown of BMI1 in irradiated GSCs led to an impaired DNA damage repair response, whereas overexpression led to increased radioresistance [86]. Collectively, BMI1 and the PRC1 complex plays important roles in GSC maintenance and act as a potential driver of radioresistance.

Sonic hedgehog (SHH) and Wnt signalling also help to maintain GSC populations [9, 87–89]. Studies carried out in Ink4A/Arf null astrocytes show that knockdown of Cul3 was sufficient to drive a GSC-type phenotype. Mechanistically, Cul3 targets Dvl2 (Wnt) and Gli2 (SHH) and they are upregulated following the reduction of Cul3 levels, leading to ligand-independent activation of Wnt and SHH signalling. Interestingly, inhibitor of differentiation 1 expression is high across glioma subtypes, whereas Cul3 is reduced, particularly in mesenchymal and classical subtypes. In addition to the roles of LZTR1 and KBTBD7 previously described, these studies suggest that the regulation of Cul3 protein levels could be important in maintaining GSCs, particularly in mesenchymal and classical subtypes [89]. RNF41 is an E3 ligase which is also downregulated in GB and can attach K63-linked ubiquitin onto Dvl2 in the presence of Vangl1/2. This linkage abrogates Dvl2 binding to phosphatidic acid, which in turn reduces the binding of Dvl2 at the frizzled receptor, resulting in reduced non-canonical Wnt signalling and the promotion of GB invasiveness and migration [90]. However, while this function of RNF41 has been evidenced in GB cells, it has yet to be shown in GSCs and additional studies are required to demonstrate its relevance in this setting.

RE1 silencing transcription factor (REST) is expressed in NSCs to prevent neural differentiation [91]. Although not frequently overexpressed in GB, REST could be critical in maintaining GSC populations [92–94]. The SCF^βTrCP^ complex modulates REST stability by tagging it for degradation via the proteasome [95, 96]. Following neuronal differentiation, there is a 13-fold increase in βTrCP levels and overexpression of βTrCP is sufficient to differentiate NSCs into neurons [96]. As REST is only expressed in GSCs rather than differentiated brain cells, increasing the activity of the SCF^βTrCP^ complex could be an important cancer-selective method of treating GB [94]. SCF^FBXL14^ also plays an important role in GSC differentiation. Non-GSCs preferentially express SCF^FBXL14^ and the complex leads to GSC differentiation via the ubiquitination and degradation of Myc. This function is opposed by the deubiquitinase ubiquitin-specific peptidase 13, which reverses SCF^FBXL14^-mediated ubiquitylation [97]. Furthermore, SCF^FBXL14^ is also an E3 for HES1, a critical component of the Notch signalling pathway and an important mediator of stemness [98]. Therefore, the ability of FBXL14 to mediate stemness in GSCs may be broader than its defined action on c-Myc; further investigation in disease-relevant models would be required to demonstrate this. The HECT, UBA and WWE domain-containing E3 ubiquitin-protein ligase 1 (Huwe1) can suppress the N-Myc-DLL3 cascade in NSCs, leading to their differentiation. In GB, Huwe1 is often downregulated and focal deletions of Huwe1 have been reported in 25% of n-Myc amplified tumours [99]. This work suggests a tumour suppressive role for Huwe1 in GSCs, however, more research is required to elucidate this fully.

The gene encoding the E3 ligase TRIM8 often undergoes hemizygous deletion in GB [100]. Nevertheless, it seems to be important for maintaining GSCs. For example, it enhances the expression of stem cell markers such as Sex determining region Y-box 2 (SOX2) and CD133 by suppressing the expression of the inhibitor of STAT3, protein inhibitor of activated STAT1 (PIAS1). Although unclear, it is believed to be through ubiquitylation and subsequent degradation of PIAS1 [101]. In addition, TRIM8 binds the DNA consensus sequences of STAT3, helping recruit it to STAT3 inducible elements [102]. Whilst TRIM8 can enhance SOX2 expression in GSCs, the APC/C^CDC20^ E3 ligase maintains GSCs via a SOX2-dependent mechanism. In patient-derived GSCs, APC/C^CDC20^﻿ stabilised SOX2 and was enriched in stem cell populations [103, 104]. Even though the direct mechanism of stabilisation is unclear, it may occur via an APC/C^CDC20^-dependent ubiquitylation of an unidentified E3 ligase that targets SOX2 for degradation [104]. Interestingly, in NSCs, the Cul4A^DET1-COP1^ complex can ubiquitylate SOX2, leading to its degradation [105]. Given the similarities between NSCs and GSCs, one could hypothesise that the Cul4A^DET1-COP1^ may be targeted by the APC/C^CDC20^ complex to maintain SOX2 stability. Finally, RNF19A was identified as an essential protein that maintains the GSC phenotype in novel 3D printed models of GB [106].

## E3 ligases—controlling cell death in GB

Apoptosis is an important mediator of cell death in cancer cells [107, 108]. Two forms of apoptosis have been described, namely the intrinsic and extrinsic pathways. Inhibitors of apoptosis (IAPs) are E3 ligases that are intimately involved in the regulation of cell death. In particular, cIAP1 (BIRC2), cIAP2 (BIRC3) and X-linked IAP (XIAP) have been extensively studied and are therapeutic targets in GB [109].

The most well-defined functions of BIRC2/3 occur during tumour necrosis factor receptor 1 (TNFR1) signalling. Upon binding its cognate ligand (TNF-α or lymphotoxin-α), trimerization of TNFR1 leads to the recruitment of TNF receptor type 1-associated death domain (TRADD) protein which acts as a scaffold facilitating the recruitment of several proteins including the E3 ligases TRAF2, BIRC2 and BIRC3 to form complex I [110–114]. This complex includes RIPK1, which undergoes BIRC2- and BIRC3-mediated K11, K48 and K68 linked ubiquitylation [115–118]. These polyubiquitylation events lead to the recruitment of the linear ubiquitin assembly complex which contains the E3 ligase HOIL 1-interacting protein (HOIP). HOIP can attach linear ubiquitin chains which stabilise the TNFR signalling complex and recruit other factors such as NFκB essential modulator (NEMO) leading to the activation of NFκB [119]. The NFκB signalling pathway has multiple pro-tumorigenic roles including cell proliferation, angiogenesis and maintenance of cancer stem cells [120]. RIPK1-dependent NFκB activation leads to the upregulation of Mdm2, the E3 ligase for p53 that is amplified or mutated in 10% of GB. Levels of RIPK1 and Mdm2 strongly correlate in GB and high RIPK1 expression confers poorer prognosis on patients [9, 121]. Targeting Mdm2 is indeed a promising strategy for treating p53 wild-type GB tumours, although getting sufficient amounts of therapeutics across the blood-brain barrier remains a challenge [122–125]. As it is altered in ~85% of GB cases [9], the p53 pathway is a critical regulator of GB development. For a comprehensive overview of this, we would direct readers to recent reviews [125].

If the E3 ligase activity of BIRC2/3 is absent, RIPK1/TRADD dissociate from TNFR1 and form a cytosolic complex, termed complex II, which contains the death-inducing signalling complex (DISC) proteins procaspase 8, FADD and FLIP [112]. The composition of this complex is critical. For example, caspase 8 and the FLIP isoform FLIP_(L)_ form a spatially restricted, enzymatically active heterodimer which can cleave RIPK3 leading to inhibition of cell death via necroptosis. In contrast, if FLIP is absent, caspase 8 is fully activated and apoptosis is induced. Importantly, BIRC2/3 act as arbiters between pro-survival and pro-death signalling in this setting.

Second mitochondria-derived activator of caspase (SMAC) mimetics mimic the N-terminal AVPI motif of SMAC/Direct IAP-Binding protein with Low PI (Diablo) leading to autoubiquitylation and degradation BIRC2/3 and direct disruption of their ubiquitin signalling complexes. Stem cells derived from GB and primary glioma cells treated with SMAC mimetics undergo NFκB-dependent apoptosis and their use is highly synergistic with γ-irradiation [126]. The radiosensitisation effect observed with SMAC mimetics is also observed upon silencing of TRAF2, which inhibits GB cell growth in an NFκB-dependent manner [127]. XIAP also moderates the resistance of cells to γ-irradiation and the combination of XIAP inhibitors with γ-irradiation sensitises GSCs and primary GB cells to apoptosis while maintaining normal neuron viability [128]. A study combining the SMAC mimetic BV6 with TMZ also observed highly synergistic induction of RIPK1 and NFκB-dependent cell death in GB cells [129], although patient-derived cells showed varied responses to TMZ and SMAC mimetic drug combinations [130]. Also, combination of SMAC mimetics with immune checkpoint inhibitors was reported to achieve 100% durable response rate in orthotopic models of GB, with tumours killed in a CD8^+^ T cell- and TNFα-dependent manner [131]. Taken together, these studies demonstrate that IAPs are critical mediators of cell death and bona fide therapeutic targets for GB.

In GB, TRAF2- and BIRC3-dependent signalling platforms are present independently of TNFR1. For example, they are recruited to the mutant EGFRvIII receptor whereby they attach K63-linked ubiquitin to RIPK1, leading to downstream NFκB activation. Interestingly, the EGFR WT receptor acts as a negative regulator of this complex and can drive cell death via the RIPK1-dependent ripoptosome [132, 133]. Treatment of EGFR WT cells co-expressing EGFRvIII with EGF dissociates the E3 ligase-RIP complex, resulting in cell death. Critically, loss of RIPK1 abrogates the tumour-forming capability of these cells in an in vivo orthotopic model of GB. These results suggest that the oncogenicity of the EGFRvIII mutation in GB may require the recruitment of the E3 ligase-RIP1 complex [133].

The A20 protein is unique in that it can act as a deubiquitinase (DUB) via its n-terminal ovarian tumour domain and as an E3 ligase via its zinc finger 4 (ZN4) domain. A20 can bind to the TNFR1 complex I and use its DUB activity to remove ubiquitin, destabilise the complex and reduce pro-survival NFκB signalling [134]. Alongside FBXO32, A20 is also a key mediator of NFκB-mediated resistance to BCNU and TMZ treatment [135] and is a target for miR-125b which prevents TMZ resistance in GB models [136]. Additionally, GB cell lines and GSCs enriched from patients express high levels of A20 compared to normal tissue. The expression of CD133, a GSC marker, and A20 correlated; silencing of A20 led to cell death in patient-derived GSCs. Knockdown of A20 also attenuated neurosphere formation in GSCs and prevented tumour development in xenograft models. Furthermore, A20 is elevated in glioma, enriched in mesenchymal subgroups (especially with *TP53* and *NF1* mutations) and its expression correlates with poor survival [137]. A20 is also reported to mediate TRAIL-R2 signalling, which is being studied as a therapeutic target due to the cancer cell-specific death induced by its activation [138, 139]. Activation of TRAIL-R2 with its ligand TRAIL leads to the formation of DISC, caspase 8 activation and cell death via apoptosis. A20 can inhibit this signalling via ZN4-dependent K48- and K63-mediated ubiquitylation of RIPK1. The K63-linked chains on RIPK1 bind directly to caspase 8, reducing its cleavage and activation. In TRAIL-resistant GB cells, a pre-ligand assembly complex (PLAC) comprising of TRAF2, A20, TRAIL-R2 and RIPK1 was identified. PLAC in TRAIL-sensitive cell lines lacked A20 and RIPK1, a feature also observed in GSCs. Therefore, A20’s E3 ligase activity is a critical determinant of TRAIL sensitivity in GB [139].

In cell models of GB, low levels of PTEN resulted in high levels of p-Akt which, in turn, increased the polyubiquitylation of the E3 ligase ITCH. ITCH is an E3 ligase for the DISC protein, FLIP_(s)_. Through stabilisation of FLIP_(S)_, PTEN deficient GB cells can become resistant to TRAIL-induced apoptosis [140, 141]. In non-GB models, activation of TRAIL-R2 DISC promoted Cul3-dependent polyubiquitylation of caspase 8 and enhanced cell death. A20 reversed this phenotype via caspase 8 deubiquitylation [142]. Recently, it has also been reported that the SCF^Skp2^ complex can alter the levels of the endogenous inhibitor/activator of caspase 8, FLIP_(L)_, at the DISC, the levels of which are critical for determining cell death or survival [143, 144]. Whether similar regulation occurs in GB is yet to be shown but these data demonstrate the complex interplay between E3 ligases that determines a cell’s sensitivity to death receptor-mediated apoptosis.

In around 45% of cases, GB tumours exhibit loss of chromosome 9p21, which confers a survival advantage primarily through the deletion of the *CDKN2A* locus [1]. The gene encoding the metabolic enzyme 5-methylthioadenosine phosphorylase (MTAP) is also located at this locus and is often co-deleted [145]. MTAP converts 5-methylthioadenosine (MTA), a by-product of the polyamine biosynthesis pathway, to 5-methylthioribose 1-phosphate, which is further processed to generate methionine and adenine via the methionine salvage pathway [146]. A recent study demonstrated that loss of MTAP can lead to increased levels of MTA which can inhibit the activity of protein arginine methyltransferase 5 (PRMT5) [147]. In GB, it has been reported that basal expression of RNF168, an E3 ligase that has essential roles in double-strand break repair following IR [148, 149], is controlled by PRMT5/PRMT7 [150]. The study demonstrated that deletion of MTAP is sufficient to cause a reduction in RNF168 expression and sensitise GB cells to IR [150]. Mechanistically, RNF168 activity modifies H2AX, a histone marker essential in the recruitment of DNA double-strand break repair proteins to areas of DNA damage. The activity of RNF168 stabilises H2AX via ubiquitination on K13 and K15, preventing SMURF2-mediated K119 ubiquitination and degradation. In MTAP deficient tumours, the dynamic between RNF168 and SMURF2 is altered and therefore, H2AX is destabilised and GB cells sensitised to IR [150]. Furthermore, in radioresistant GSCs, the lipolytic inhibitor G0/G1 switch gene 2 (GOS2) is upregulated and promotes mTOR/S6K activation. This, in turn, reduces RNF168 expression and enhances the resistance of GSCs to radiation [151]. Collectively, these studies identify a crucial role for RNF168 in mediating IR sensitivity in GB.

In normal brain tissue, the E3 ligase RNF138 is localised to the endoplasmic reticulum but translocates to the nucleus in glioma [152]. Within the nucleus, RNF138 can ubiquitylate ribosomal protein S3 (rpS3) on K214, leading to its degradation via the proteasome. rpS3 interacts with DNA damage-inducible transcript 3, leading to the expression of the pro-apoptotic protein growth arrest and DNA damage-inducible 34 (GADD34). Following RNF138-mediated degradation of rpS3, GADD34 expression is reduced and GB cells become more resistant to IR via a reduction in apoptosis [153].

Finally, increases in oxidative stress cause the migration of pyruvate kinase M2 to the mitochondria where it can phosphorylate the anti-apoptotic protein B-cell lymphoma 2 (Bcl-2) on T69 [154]. Bcl-2 prevents mitochondrial outer-membrane permeabilisation (MOMP) and inhibits intrinsic apoptosis [155]. This phosphorylation prevents Cul3–RBX1-mediated degradation of Bcl-2, protecting GB cells from oxidative stress-induced apoptosis [154].

**Fig. 4:**
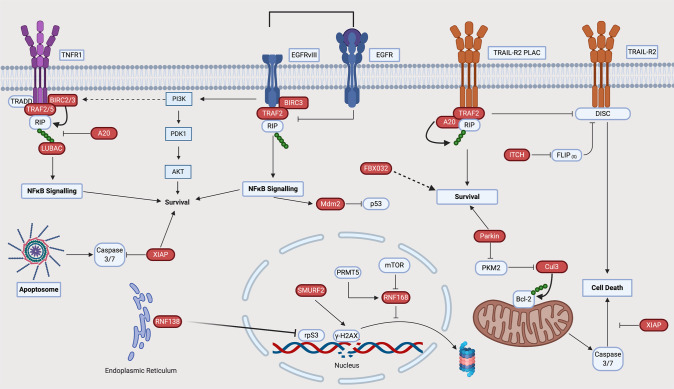
The role of E3 ubiquitin ligases in GB cell death. The goal of therapeutic intervention in cancer is to kill or remove tumour cell populations. At the receptor level, E3 ligases form multi-component complexes to moderate cell death signalling from death receptors such as TRAIL-R2. In addition, similar complexes can moderate dichotomous outcomes (death or survival) from TNFR1 and EGFRvIII receptor activation. Downstream of this, E3 ligases also moderate DNA damage responses and intrinsic apoptosis activation. Red icons represent E3 ligases and their functions as described in GB models.

The topics discussed in this section of the review are summarised in Fig. 4 and Table 3.

**Table 3:** E3 ubiquitin ligase-dependent regulation of cell death in glioblastoma.

E3 ligase	Substrate/Downstream effector	Pathway	Reference(s)
A20	RIPK1	TNF-α signalling, EGFR signalling, death receptor-mediated apoptosis, stem cell maintenance	[134–137, 139, 164]
BIRC2	RIPK1	TNF-α signalling	[118, 129, 165, 166]
BIRC3	RIPK1	TNF-α signalling	[116, 118, 132, 167]
Cul3	BCL-2	Intrinsic apoptosis	[154]
HOIP	–	TNF-α signalling	[119]
ITCH	FLIP_(S)_	Death receptor-mediated apoptosis	[140, 141]
Mdm2	p53	Intrinsic apoptosis	[122–125]
RNF138	rpS3	DNA damage repair/apoptosis	[153]
RNF168	H2AX	DNA damage repair	[148–151]
SMURF2	H2AX	DNA damage repair	[150]
TRAF2	–	TNF-α signalling, EGFR signalling, death receptor-mediated apoptosis	[110, 112, 114, 127]
XIAP	Caspase 3/7/9, survivin	Intrinsic apoptosis	[128, 167–171]

## Conclusions and future perspectives

Tumours present as heterogeneous diseases and are layered with complexity at the genetic and epigenetic level. Beyond simple genetic mutation and amplification, the post-transcriptional modification of proteins is now being revealed on a larger scale with technologies such as reverse phase protein lysate microarrays. Within this complex milieu, E3 ubiquitin ligases can alter tumour-critical functions such as oncogenic protein stability, protein localisation, cell death, gene transcription and pro-survival signalling making them a central cog in promoting the cancer hallmarks. With >700 E3 ligases in the human genome, significant gaps in our knowledge remain and need to be addressed before we can exploit the full power of these proteins as cancer therapeutics. Principle among these is the need to understand how E3 ligases identify their specific substrates as only a handful of recognition motifs, termed degrons, have been identified thus far. Further, as demonstrated by β-Trcp1/2, there is significant redundancy in the substrate recognition system, meaning that identification of substrates unique to each E3 ligase may be required to facilitate efficient small-molecule inhibition of substrate degradation. Finally, and as highlighted in this review, E3 ligases exhibit distinct functions dependent on tumour types, suggesting a tumour-specific approach to E3 ligase biology will be fruitful going forward. Beyond small-molecule inhibitors, proteolysis targeting chimeras (PROTACs) have emerged as significant future therapeutic opportunities in the E3 ligase field. PROTACs induce proximity between an E3 ligase and a protein of interest (for example, an oncoprotein) to facilitate the ubiquitylation and degradation of the protein of interest. Central to this technology is identifying a specific degron to which a chosen E3 ligase will bind, an area requiring significant focus going forward to expedite the translation of this technology into the clinical setting. This class of therapeutics is particularly relevant for GB given the  IMiD drug class (thalidomide, lenalidomide and pomalidomide) of PROTACs can successfully cross the blood–brain barrier, which has been a significant challenge for effective targeting of GB with chemotherapies so far.

## Supplementary information

Supplementary information
