# Supplementary material for: The role of E3 ubiquitin ligases in the development and progression of glioblastoma
Source: Cell Death Differ. 2021 Jan 11;28(2):522–37. doi: 10.1038/s41418-020-00696-6 (PMC7862665; doi:10.1038/s41418-020-00696-6)
Supplement: Supplementary file 1 — Supplementary information [file 41418_2020_696_MOESM1_ESM.pdf]

The role of E3 ubiquitin ligases in the development and progression of glioblastoma – Supplementary Material

Luke M Humphreys✉[1], Paul Smith[1], Zhuoyao Chen[1], Shahd Fouad[1], Vincenzo D'Angiolella✉[1]

[1.] *Department of Oncology, Medical Research Institute for Radiation Oncology, University of Oxford, Oxford, United Kingdom*

✉Corresponding Authors

Vincenzo D'Angiolella: vincenzo.dangiolella@oncology.ox.ac.uk

Luke M Humphreys: luke.humphreys@oncology.ox.ac.uk

Supplementary Text 1: Histology of GB

GB presents as a heterogeneous tumor with several microscopic and molecular phenotypes used to diagnose the stage of the malignancy. Spatial distribution of the tumor is not restricted to a single region of the brain, with 43% of GB lesions occurring in the frontal lobe, 28% in the temporal lobe and 25% in the parietal lobe, alongside rare instances in the occipital lobe (3%) and rarer still in the cerebellum (1). Traditionally, GB was identified microscopically as tumors that have regions of hypercellularity, nuclear atypia, mitotic figures (e.g. abnormally distributed chromosomes) and display evidence of angiogenesis and/or necrosis (2, 3). In addition, the WHO classification of tumors of the CNS (2007) also describes endothelial proliferation and glomeruloid microvascular proliferation as histological features of GB (3). The 2016 WHO classification of CNS tumors represented a shift in the staging of astrocytic tumors from traditional histological phenotypes, to include common molecular alterations found in CNS tumours (4, 5). For example, IDH status (mutant or wild-type) became the first molecular classifier of the disease. Furthermore, a new variant of the disease (termed epitheloid

GB) was described which presents with Serine/threonine-protein kinase B-raf (*BRAF)* V600E mutations. Epitheloid GB is found in younger patients and, unlike many GB cases, is not associated with epidermal growth factor (*EGFR)* amplification and chromosome 10 loss (5). These molecular demarcations of GB demonstrate a shift to an improved integrated staging assessment of CNS tumours going forward and highlight the importance of developing our molecular understanding of the disease. A methylator phenotype, termed Glioblastoma – CpG Island Methylator phenotype (G-CIMP^{+}) has also been described (6). These tumours are mostly proneural (88%), occur in younger patients (average age of 36 vs. 59 when compared to G-CIMP^{-} patients who harbour proneural tumours) and convey a significant survival advantage when compared to proneural G-CIMP^{-} tumours (150 weeks vs. 42 weeks, respectively)(6).

Importantly, GB molecular subtypes are not fixed and recurrent tumours can switch between subtypes (6, 7). Single-cell transcriptomic analysis of GB tumours revealed that all four cellular subtypes are present in every tumor (8). The situation is further complicated by the presence of a hierarchy of cells in GB. Within the tumor population are a small subset of cells that are known as glioma stem cells (GSCs). GSCs are capable of forming tumours *in vivo* and demonstrate key stem cell features such as self-renewal, differentiation and neurosphere generation *in vitro* (9, 10). Likely, the GSC population provides a renewable pool of cellular states (11). Thus, while the molecular classification in GB is important to define the underlying biological pathways that are altered, it would require further integration and validation before being used to direct specific treatments.

Supplementary Text 2: Molecular Classification of GB

Several studies have collectively classified GB into 3-4 molecular subtypes (6, 12-14). Integrated genomic analysis of ~200 GB samples identified 4 subtypes of GB, termed Classical,

Mesenchymal, Proneural, and Neural. Classical GB is defined by *EGFR* amplification (97% of cases), whilst Mesenchymal subtypes predominantly have mutations in the RAS GTPase activating protein (GAP) neurofibromatosis 1 (*NF1)* gene. Proneural cases are defined by *PDFGRA* amplification with *IDH1* mutations. Whilst four original classifications were identified, it was later suggested that the neural subtype may have been identified due to contamination of the samples with non-tumour cells (7). Importantly, these classifications potentially have clinical relevance, as intensive therapy improves survival in patients with classical GB compared with those harbouring proneural tumors GB (14).

Supplementary Text 3: Overview of E3 Ubiquitin Ligases

- Ubiquitin (Ub) is covalently attached to proteins via an enzyme cascade involving a ubiquitin-activating enzyme (E1), a ubiquitin-conjugating enzyme (E2), and a ubiquitin ligase (E3).
- Substrates can be mono- or polyubiquitylated
- Ubiquitylation acts as a signal, determining the fates of modified substrates [Figure 1] (15, 16).
- There are >700 E3s (17, 18) in the human genome, which are classified into two types: **R**eally **I**nteresting **N**ew **G**ene (RING) and **H**omologous to the **E**6AP **C**arboxyl **T**erminus (HECT) [Figure 1].
- Deubiquitinases (DUBS) can reverse ubiquitylation. The majority of DUBs contain an active site, which may be targeted by small-molecule inhibitors(19). The biology and therapeutic targeting of DUBs have been recently reviewed (19, 20).

RING E3s are characterized by their zinc-bound RING catalytic domain, which recruits E2-Ub conjugates and enables direct Ub transfer from E2 to a substrate protein (21). Among the

RING E3s, the cullin-ring ubiquitin ligase (CRL) family is the largest (22). CRLs utilize cullin proteins as a central scaffold which binds to a RING-box protein and an adaptor protein–substrate receptor complex through its C- and N-termini, respectively (23). CRL1s, more commonly known as SCF (Skp1–Cul1–F-box) complexes, are proto-typical CRLs. They utilize a toolbox of ~70 F-box proteins, which Cul1 interacts with via Skp1, to recruit substrates. Whilst the organization of other CRLs resembles that of CRL1s, each CRL sub-family has a distinct set of adaptors and substrate recruiters. The structurally similar Cul2 and Cul5 employ VHL (von Hippel-Lindau)- and SOCS (suppressor of cytokine signaling)-box proteins, respectively, as substrate receptors via the adaptor complex Elongin B/C (24). CRL3s use BTB (broad complex, tramtrack and bric-à-brac)-domain proteins as both adaptors and substrate receptors, which recognize substrates using their meprin and TRAF homology (MATH) motif or Kelch beta-propeller repeats (25, 26). The 82% identical Cul4A and Cul4B use DNA damage-binding protein 1 (Ddb1) as an adaptor, which in turn binds to a sizeable family of substrate recruitment subunits collectively called Ddb1- and Cul4-associated factors (DCAFs) (27). The most recently identified members of the cullin family, Cul7 and Cul9, happen to be the largest and yet least related to the other cullins (28, 29). Similarly to Cul1, Cul7 binds to Skp1 and the F-box protein Fbxw8, however, it does not interact with other F-box proteins (28). Attributes of a CRL assembly for Cul9 are yet to be elucidated.

A similar mechanism and folding structure to the RING domain is employed by U-box E3 ligases, which are also categorized as RING-type E3s. However, their zinc-bound sites are replaced by a hydrophobic core to support protein folding (30). By contrast, HECT E3s undergo a catalytic cysteine-dependent trans-thiolation reaction with E2-Ub, producing a transitory E3-Ub whose formation precedes Ub transfer to the substrate (31). A third and emerging class of E3s, RING-IBR-RING (RBR), is a hybrid of RING-HECT E3 subtypes (32, 33). The RBR protein comprises two RING fingers, RING1 and RING2, and an in-between-

RINGs (IBR) domain. The RING1 domain, similar to classic RING-type E3s, binds to E2-Ub and brings it into close proximity with the RING2 domain, in which a conserved cysteine is capable of forming a reversible thioester intermediate with Ub (32, 33). More recently, a fourth derivative class called RING-Cys-Relay (RCR) has been reported following a study on its member MYC Binding Protein 2 (MYCBP2). In this model, two catalytic cysteines mediate intramolecular ubiquitin relay to achieve steric preference for non-lysine substrate ubiquitylation (34).

Supplementary Text 4: RTK signalling – Nucleus

RTK signalling results in altered transcriptional profiles that ultimately coordinate cellular responses with extracellular stimuli. A modulator of EGFR-dependent Ras signalling is the Capicua (CIC) protein, which represses the activity of several transcription factors operating downstream of Ras (35). CIC levels are reduced in GB resulting in the upregulation of repressed targets of RTK signalling such as ETS Variant Transcription Factor 1 (ETV1)(36). CIC can also suppress Ras-mediated proliferation and deletion of CIC in murine stem cells increases neurosphere formation, indicating that CIC also represses stemness. The stability of CIC is regulated by Erk-dependent phosphorylation on S173 which permits Praja1-mediated ubiquitination and degradation of CIC in GB (36). In *drosophila,* a mutant version of the COP9 signalosome subunit 1b reduces ectopic, EGFR-dependent gene expression by protecting CIC from ubiquitylation and degradation. It was also shown that CIC degradation was dependent on a Cullin 1/SKP1-related A/Archipelago E3 ligase complex suggesting that multiple E3 ligase complexes may be involved in the regulation of CIC (37).

E3 ligases can also influence RTK signalling independently of ubiquitylation. For example, EGFR signalling can lead to non-canonical H3K23Ac which can be recognised and bound by the E3 ligase TRIM24. TRIM24 can recruit STAT3, an oncogenic transcriptional activator, and enhance downstream signalling (38). Importantly, the ability of TRIM24 to modulate STAT3 signalling is independent of its RING domain (38). Therefore, the function of E3 ligases as adaptor/scaffold proteins must also be considered in addition to their canonical role in ubiquitylation.

Supplementary Text 5: Cancer signaling pathways beyond RTKs

The topics discussed in this section are summarised in supplementary figure 1 and supplementary table 1.

The Hippo signalling pathway is frequently altered in cancer and mutations in this pathway have been identified in a small number of GB cases (39). Components of the pathway, such as the MOB kinase activator 1 (MOB1), which is part of the nuclear-Dbf2-related kinase 1 (NDR) /Large Tumour Suppressor kinase (LATS) are modified post-transcriptionally in GB models (40). The NDR/LATS complex phosphorylates Yes-associated Protein (YAP), a transcriptional co-activator and oncoprotein, on S127. Once phosphorylated, YAP is sequestered in the cytoplasm by 14-3-3 proteins and degraded (41, 42). The E3 ligase Praja2 can target MOB1 for degradation resulting in reduced YAP phosphorylation. YAP can translocate into the nucleus where it promotes the expression of anti-apoptotic and pro-proliferative genes (40). In support of this, Praja2 maintains GB cell line growth *in vivo* and is overexpressed in GB patient samples (40). YAP is also a target for the $SCF^{\beta\text{-}Trcp}$ complex (42). In GB, the activity of $SCF^{\beta\text{-}TrCP}$ can be thwarted through the binding of Actin-like 6A (ACT6LA) to YAP, which prevents YAP degradation and thereby facilitates oncogenesis(43). Taken together, these findings

suggest that E3 ligases could have important roles in the post-translational regulation of the tumour suppressive Hippo pathway in GB.

Transforming growth factor-β (TGF-β) signalling is also regulated by E3 ligases in GB. Upon activation, TGF-β receptors can phosphorylate signal transduction proteins known as receptor-regulated SMADs (rSMADs), which can form heteromeric complexes with SMAD4 and translocate to the nucleus leading to alterations in gene expression. The downstream signalling from TGF-β receptors can be negatively regulated by the inhibitory SMAD6 and SMAD7 (44). SMAD-Specific E3 Ubiquitin Protein Ligase 2 (SMURF2), an E3 ligase of the C2-WW-HECT domain class, can bind SMAD7 and directly ubiquitinate the TGF-β receptor via the recruitment of the E2 UbcH7 (45, 46). Ubiquitin specific peptidase (USP15) is also able to bind SMAD7 and stabilise the TGF-β receptor via the removal of ubiquitin. In GB models, the balance of activity between USP15 and SMURF2 can regulate TGF-β signalling. Overexpression of USP15 has been reported in GB and loss of USP15 in GSCs is sufficient to reduce the size of intracranial tumours *in vivo* (47). The HECT and RLD Domain Containing E3 Ubiquitin Protein Ligase 3 (HERC3) can also act as a positive regulator of TGF-β signalling by translocating SMAD7 from the nucleus to the cytoplasm and attaching K63 linked polyubiquitin chains. Once ubiquitinated, SMAD7 is degraded via the autolysosome pathway (48). Furthermore, high levels of HERC3 correlate with low levels of SMAD7 expression and poor prognosis in classical GB, suggesting that the HERC3/SMAD7 axis could be important in determining patient outcomes in GB (48).

GB is characterised by areas of hypoxia and the turnover of proteins involved the hypoxia response can be modulated by E3 ligases (49). A well-established role of E3-ligases is that of the Elongin B/C-Cul2-Von Hippel Lindau (VCB) complex, which degrades the Hypoxia

Inducible Factor 1 Subunit Alpha (HIF1α) transcription factor in normoxic conditions (50). During periods of hypoxia, HIF1α is no longer degraded and associates with HIF1β to drive alterations in gene expression (50-53). In GB, tumour-derived angiogenic factors promote Src and vascular endothelial growth factor receptor 2-dependent phosphorylation of the cytoskeletal binding protein profilin-1 (PFN-1) on Y129. Once phosphorylated, PFN-1 can bind to the VCB complex leading to a reduction of HIF1α turnover. The reduction in VCB activity leads to blood vessel growth and GB development (54). In addition to the VCB complex, the E3 ligase Mdm2 is known to degrade HIF1α during hypoxia, a function that is attenuated in the presence of active PI3K/Akt signalling (55). In GSCs, the VCB complex can modulate levels of HIF2α in a dual-specificity tyrosine phosphorylation regulated kinase 1A (DYRK1A)-dependent manner. In the absence of DYRK1A phosphorylation, Inhibitor of differentiation 2 (ID2) protein can bind to Cul2 and dissociate it from the VCB complex. This leads to HIF2α stabilisation and maintenance of the GSC phenotype (56). E3 ligases, therefore, play a critical tumour suppressor role in the hypoxia environments observed in GB.

Recently, the RNA binding E3 ligase Mex3A was identified as a potential therapeutic target in GB. Although the substrates for this E3 ligase are not well understood, recent work has uncovered retinoic acid-inducible gene-1 (RIG-1) as a target of Mex3A, causing RIG-1 degradation. Interestingly, Mex3A is overexpressed in GB and silencing of Mex3A caused a reduction in GB cell proliferation and migration (57). RIG1 is a known suppressor of β-catenin signalling via NFκB activation (58). β-catenin is a key component of the Wnt signalling cascade, is important in cell adhesion and has been implicated in GB development and progression(59). Several studies have described $SCF^{\beta TrCP}$ as an E3 ligase targeting n-terminal phosphorylated β-catenin for degradation and Cbl targets nuclear β-catenin for degradation (60-63). In GB models, TRIM33 can initiate K48-dependent degradation of β-catenin-PSer715

and does not require the n-terminal phosphorylation required by $SCF^{\beta TrcP}$. Loss of TRIM33 leads to the significant proliferation of tumours in orthotopic U87 mouse models and reduced levels of TRIM33 are observed in GB samples (64).

Finally, Myc is a transcription factor that is commonly overexpressed/amplified in GB (particularly in G-CIMP^{+} tumours) and whose stability is regulated by E3 ligases (65, 66). Functional network analysis identified two E3 ligases, F-box and WD repeat domain containing 7 (FBXW7) and F-box only protein 2, who are binding partners of Myc and are under-expressed in gliomas. From these, FBXW7 was confirmed as a *bona fide* E3 ligase for Myc (66, 67). The expression of *FBXW7* is reduced in 80% of GB samples when compared to normal controls and overexpression of FBXW7 is sufficient to inhibit the proliferation of model GB cell lines (68). Interestingly, TRIP13 can stabilise Myc in GB via its ability to negatively regulate the FBXW7 promoter, which in turn drives the proliferation, invasion and the migration of GB cells (69). Circular RNA forms of FBXW7 have also been identified as prognostic indicators in GB and can repress GB growth *in vivo* (70). FBXW7 is a tumour suppressor in multiple tumour types (71) and these studies suggest that this is also the case in the context of GB. However, only ~1% of GB have mutations in FBXW7, meaning there are other mechanisms (beyond mutations in FBXW7) of Myc activation and /or FBXW7 inactivation in GB.

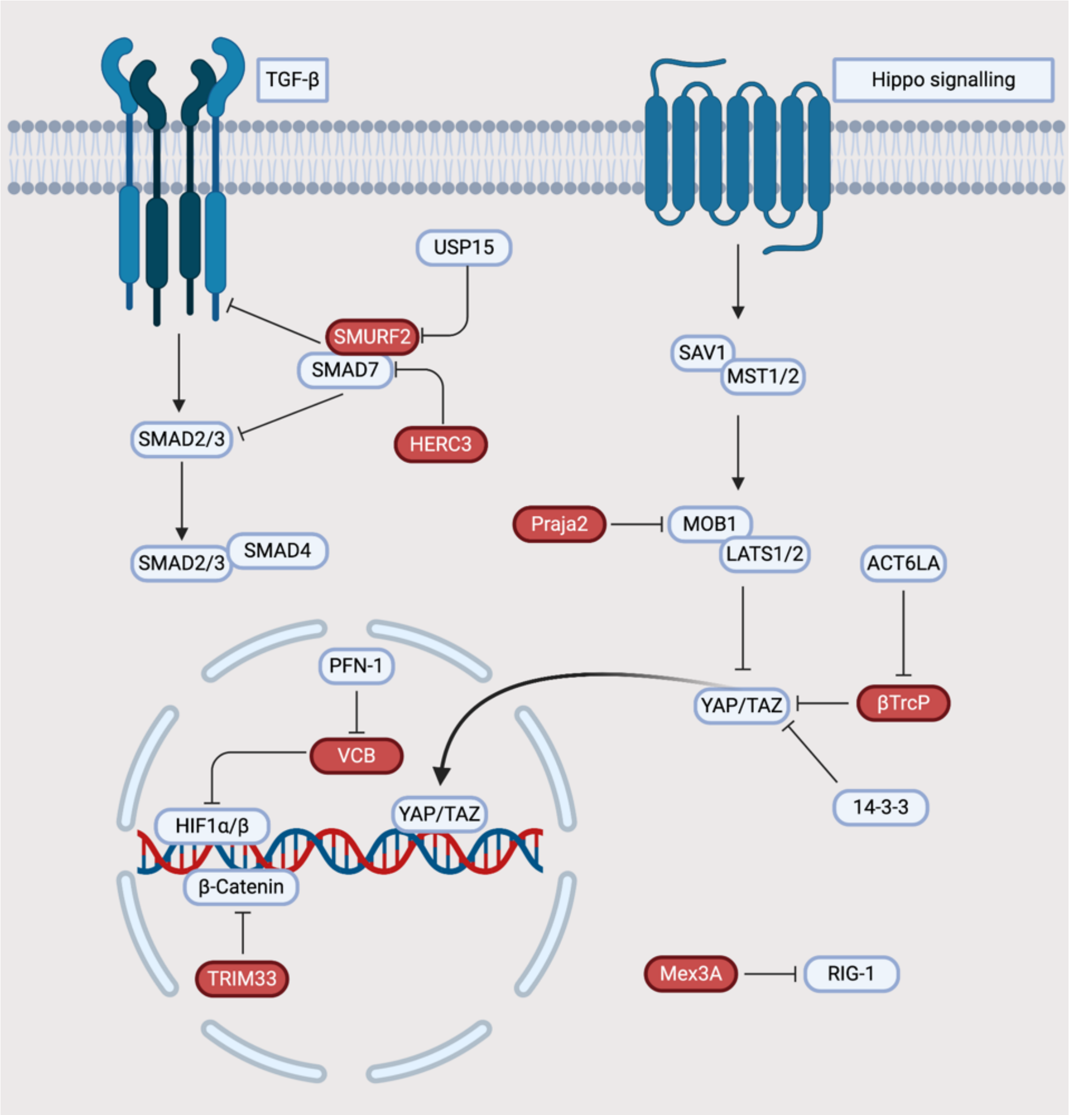

Supplementary Fig 1: Cancer Signalling pathways beyond RTKs

At the receptor level, E3 ligases interact with TGF-β in contrasting roles, both in the promotion and inhibition of this pathway. The tumor suppressive hippo signalling pathway is also regulated by E3 ligases, controlling the translocation of oncogenic YAP/TAZ transcription factor into the nucleus. Other signalling pathways, such as hypoxia (HIF1α/β) and β-catenin-dependent transcription, are also regulated by E3s. *Red icons represent E3 ligases and their functions as observed in GB models.*

Supplementary Table 1 E3 ubiquitin ligase-dependent Modulation of Cancer signalling pathways beyond RTKs in Glioblastoma

E3 Ligase	Substrate/ Downstream effector	Pathway	Reference(s)
FBXW7	MYC	-	(67-70)
HERC3	SMAD7	TGF-β	(48)
MDM2	HIF1α	Hypoxia response	(55)
MEX3A	RIG-1	NFκB	(57)
PRAJA2	MOB1	Hippo	(40)
$SCF^{\beta TrCP}$	YAP	Hippo	(42, 43)
SMURF2	TGF-β Receptor	TGF-β	(45)
TRIM33	β-catenin	Wnt	(64)
VCB Complex	HIF1/2α	Hypoxia response and stem cell maintenance	(50, 54, 56)

Supplementary References

1. Simpson JR, Horton J, Scott C, Curran WJ, Rubin P, Fischbach J, et al. Influence of location and extent of surgical resection on survival of patients with glioblastoma multiforme: results of three consecutive Radiation Therapy Oncology Group (RTOG) clinical trials. Int J Radiat Oncol Biol Phys. 1993;26(2):239-44.
2. Gladson CL, Prayson RA, Liu WM. The pathobiology of glioma tumors. Annu Rev Pathol. 2010;5:33-50.
3. Louis DN, Ohgaki H, Wiestler OD, Cavenee WK, Burger PC, Jouvet A, et al. The 2007 WHO classification of tumours of the central nervous system. Acta Neuropathol. 2007;114(2):97-109.
4. Molinaro AM, Taylor JW, Wiencke JK, Wrensch MR. Genetic and molecular epidemiology of adult diffuse glioma. Nat Rev Neurol. 2019;15(7):405-17.
5. Louis DN, Perry A, Reifenberger G, von Deimling A, Figarella-Branger D, Cavenee WK, et al. The 2016 World Health Organization Classification of Tumors of the Central Nervous System: a summary. Acta Neuropathol. 2016;131(6):803-20.
6. Noushmehr H, Weisenberger DJ, Diefes K, Phillips HS, Pujara K, Berman BP, et al. Identification of a CpG island methylator phenotype that defines a distinct subgroup of glioma. Cancer Cell. 2010;17(5):510-22.
7. Wang Q, Hu B, Hu X, Kim H, Squatrito M, Scarpace L, et al. Tumor Evolution of Glioma-Intrinsic Gene Expression Subtypes Associates with Immunological Changes in the Microenvironment. Cancer Cell. 2017;32(1):42-56 e6.
8. Neftel C, Laffy J, Filbin MG, Hara T, Shore ME, Rahme GJ, et al. An Integrative Model of Cellular States, Plasticity, and Genetics for Glioblastoma. Cell. 2019;178(4):835-49 e21.
9. Robertson FL, Marques-Torrejon MA, Morrison GM, Pollard SM. Experimental models and tools to tackle glioblastoma. Dis Model Mech. 2019;12(9).
10. Prager BC, Bhargava S, Mahadev V, Hubert CG, Rich JN. Glioblastoma Stem Cells: Driving Resilience through Chaos. Trends Cancer. 2020;6(3):223-35.
11. Suva ML, Tirosh I. The Glioma Stem Cell Model in the Era of Single-Cell Genomics. Cancer Cell. 2020;37(5):630-6.
12. Phillips HS, Kharbanda S, Chen R, Forrest WF, Soriano RH, Wu TD, et al. Molecular subclasses of high-grade glioma predict prognosis, delineate a pattern of disease progression, and resemble stages in neurogenesis. Cancer Cell. 2006;9(3):157-73.
13. Wang Q, Hu B, Hu X, Kim H, Squatrito M, Scarpace L, et al. Tumor Evolution of Glioma-Intrinsic Gene Expression Subtypes Associates with Immunological Changes in the Microenvironment. Cancer Cell. 2018;33(1):152.
14. Verhaak RG, Hoadley KA, Purdom E, Wang V, Qi Y, Wilkerson MD, et al. Integrated genomic analysis identifies clinically relevant subtypes of glioblastoma characterized by abnormalities in PDGFRA, IDH1, EGFR, and NF1. Cancer Cell. 2010;17(1):98-110.
15. Komander D, Rape M. The ubiquitin code. Annu Rev Biochem. 2012;81:203-29.
16. Yau R, Rape M. The increasing complexity of the ubiquitin code. Nat Cell Biol. 2016;18(6):579-86.
17. Stewart MD, Ritterhoff T, Klevit RE, Brzovic PS. E2 enzymes: more than just middle men. Cell Res. 2016;26(4):423-40.
18. Zheng N, Shabek N. Ubiquitin Ligases: Structure, Function, and Regulation. Annu Rev Biochem. 2017;86:129-57.
19. Clague MJ, Urbe S, Komander D. Breaking the chains: deubiquitylating enzyme specificity begets function. Nat Rev Mol Cell Biol. 2019;20(6):338-52.
20. Harrigan JA, Jacq X, Martin NM, Jackson SP. Deubiquitylating enzymes and drug discovery: emerging opportunities. Nat Rev Drug Discov. 2018;17(1):57-78.

21. Deshaies RJ, Joazeiro CAP. RING Domain E3 Ubiquitin Ligases. Annual Review of Biochemistry. 2009;78(1):399-434.
22. Petroski MD, Deshaies RJ. Function and regulation of cullin-RING ubiquitin ligases. Nat Rev Mol Cell Biol. 2005;6(1):9-20.
23. Sarikas A, Hartmann T, Pan ZQ. The cullin protein family. Genome Biol. 2011;12(4):220.
24. Kamura T, Maenaka K, Kotoshiba S, Matsumoto M, Kohda D, Conaway RC, et al. VHL-box and SOCS-box domains determine binding specificity for Cul2-Rbx1 and Cul5-Rbx2 modules of ubiquitin ligases. Genes Dev. 2004;18(24):3055-65.
25. Pintard L, Willems A, Peter M. Cullin-based ubiquitin ligases: Cul3-BTB complexes join the family. Embo j. 2004;23(8):1681-7.
26. Xu L, Wei Y, Reboul J, Vaglio P, Shin TH, Vidal M, et al. BTB proteins are substrate-specific adaptors in an SCF-like modular ubiquitin ligase containing CUL-3. Nature. 2003;425(6955):316-21.
27. Jin J, Arias EE, Chen J, Harper JW, Walter JC. A family of diverse Cul4-Ddb1-interacting proteins includes Cdt2, which is required for S phase destruction of the replication factor Cdt1. Mol Cell. 2006;23(5):709-21.
28. Dias DC, Dolios G, Wang R, Pan ZQ. CUL7: A DOC domain-containing cullin selectively binds Skp1.Fbx29 to form an SCF-like complex. Proc Natl Acad Sci U S A. 2002;99(26):16601-6.
29. Marín I. Diversification of the cullin family. BMC Evol Biol. 2009;9:267.
30. Ohi MD, Vander Kooi CW, Rosenberg JA, Chazin WJ, Gould KL. Structural insights into the U-box, a domain associated with multi-ubiquitination. Nat Struct Biol. 2003;10(4):250-5.
31. Rotin D, Kumar S. Physiological functions of the HECT family of ubiquitin ligases. Nature Reviews Molecular Cell Biology. 2009;10:398.
32. Wenzel DM, Lissounov A, Brzovic PS, Klevit RE. UBCH7 reactivity profile reveals parkin and HHARI to be RING/HECT hybrids. Nature. 2011;474(7349):105-8.
33. Spratt DE, Walden H, Shaw GS. RBR E3 ubiquitin ligases: new structures, new insights, new questions. The Biochemical journal. 2014;458(3):421-37.
34. Pao KC, Wood NT, Knebel A, Rafie K, Stanley M, Mabbitt PD, et al. Activity-based E3 ligase profiling uncovers an E3 ligase with esterification activity. Nature. 2018;556(7701):381-5.
35. Jimenez G, Shvartsman SY, Paroush Z. The Capicua repressor--a general sensor of RTK signaling in development and disease. J Cell Sci. 2012;125(Pt 6):1383-91.
36. Bunda S, Heir P, Metcalf J, Li ASC, Agnihotri S, Pusch S, et al. CIC protein instability contributes to tumorigenesis in glioblastoma. Nat Commun. 2019;10(1):661.
37. Suisse A, He D, Legent K, Treisman JE. COP9 signalosome subunits protect Capicua from MAPK-dependent and -independent mechanisms of degradation. Development. 2017;144(14):2673-82.
38. Lv D, Li Y, Zhang W, Alvarez AA, Song L, Tang J, et al. TRIM24 is an oncogenic transcriptional co-activator of STAT3 in glioblastoma. Nat Commun. 2017;8(1):1454.
39. Wang Y, Xu X, Maglic D, Dill MT, Mojumdar K, Ng PK, et al. Comprehensive Molecular Characterization of the Hippo Signaling Pathway in Cancer. Cell Rep. 2018;25(5):1304-17 e5.
40. Lignitto L, Arcella A, Sepe M, Rinaldi L, Delle Donne R, Gallo A, et al. Proteolysis of MOB1 by the ubiquitin ligase praja2 attenuates Hippo signalling and supports glioblastoma growth. Nat Commun. 2013;4:1822.
41. Pan D. The hippo signaling pathway in development and cancer. Dev Cell. 2010;19(4):491-505.

42. Zhao B, Li L, Tumaneng K, Wang CY, Guan KL. A coordinated phosphorylation by Lats and CK1 regulates YAP stability through SCF(beta-TRCP). Genes Dev. 2010;24(1):72-85.
43. Ji J, Xu R, Zhang X, Han M, Xu Y, Wei Y, et al. Actin like-6A promotes glioma progression through stabilization of transcriptional regulators YAP/TAZ. Cell Death Dis. 2018;9(5):517.
44. Hata A, Chen YG. TGF-beta Signaling from Receptors to Smads. Cold Spring Harb Perspect Biol. 2016;8(9).
45. Kavsak P, Rasmussen RK, Causing CG, Bonni S, Zhu H, Thomsen GH, et al. Smad7 binds to Smurf2 to form an E3 ubiquitin ligase that targets the TGF beta receptor for degradation. Mol Cell. 2000;6(6):1365-75.
46. Ogunjimi AA, Briant DJ, Pece-Barbara N, Le Roy C, Di Guglielmo GM, Kavsak P, et al. Regulation of Smurf2 ubiquitin ligase activity by anchoring the E2 to the HECT domain. Mol Cell. 2005;19(3):297-308.
47. Eichhorn PJ, Rodon L, Gonzalez-Junca A, Dirac A, Gili M, Martinez-Saez E, et al. USP15 stabilizes TGF-beta receptor I and promotes oncogenesis through the activation of TGF-beta signaling in glioblastoma. Nat Med. 2012;18(3):429-35.
48. Li H, Li J, Chen L, Qi S, Yu S, Weng Z, et al. HERC3-Mediated SMAD7 Ubiquitination Degradation Promotes Autophagy-Induced EMT and Chemoresistance in Glioblastoma. Clin Cancer Res. 2019;25(12):3602-16.
49. Jawhari S, Ratinaud MH, Verdier M. Glioblastoma, hypoxia and autophagy: a survival-prone 'menage-a-trois'. Cell Death Dis. 2016;7(10):e2434.
50. Pause A, Lee S, Worrell RA, Chen DY, Burgess WH, Linehan WM, et al. The von Hippel-Lindau tumor-suppressor gene product forms a stable complex with human CUL-2, a member of the Cdc53 family of proteins. Proc Natl Acad Sci U S A. 1997;94(6):2156-61.
51. Lonergan KM, Iliopoulos O, Ohh M, Kamura T, Conaway RC, Conaway JW, et al. Regulation of hypoxia-inducible mRNAs by the von Hippel-Lindau tumor suppressor protein requires binding to complexes containing elongins B/C and Cul2. Mol Cell Biol. 1998;18(2):732-41.
52. Lisztwan J, Imbert G, Wirbelauer C, Gstaiger M, Krek W. The von Hippel-Lindau tumor suppressor protein is a component of an E3 ubiquitin-protein ligase activity. Genes Dev. 1999;13(14):1822-33.
53. Kamura T, Koepp DM, Conrad MN, Skowyra D, Moreland RJ, Iliopoulos O, et al. Rbx1, a component of the VHL tumor suppressor complex and SCF ubiquitin ligase. Science. 1999;284(5414):657-61.
54. Fan Y, Potdar AA, Gong Y, Eswarappa SM, Donnola S, Lathia JD, et al. Profilin-1 phosphorylation directs angiocrine expression and glioblastoma progression through HIF-1alpha accumulation. Nat Cell Biol. 2014;16(5):445-56.
55. Joshi S, Singh AR, Durden DL. MDM2 regulates hypoxic hypoxia-inducible factor 1alpha stability in an E3 ligase, proteasome, and PTEN-phosphatidylinositol 3-kinase-AKT-dependent manner. J Biol Chem. 2014;289(33):22785-97.
56. Lee SB, Frattini V, Bansal M, Castano AM, Sherman D, Hutchinson K, et al. An ID2-dependent mechanism for VHL inactivation in cancer. Nature. 2016;529(7585):172-7.
57. Bufalieri F, Caimano M, Lospinoso Severini L, Basili I, Paglia F, Sampirisi L, et al. The RNA-Binding Ubiquitin Ligase MEX3A Affects Glioblastoma Tumorigenesis by Inducing Ubiquitylation and Degradation of RIG-I. Cancers (Basel). 2020;12(2).
58. Hillesheim A, Nordhoff C, Boergeling Y, Ludwig S, Wixler V. beta-catenin promotes the type I IFN synthesis and the IFN-dependent signaling response but is suppressed by influenza A virus-induced RIG-I/NF-kappaB signaling. Cell Commun Signal. 2014;12:29.

59. Paul I, Bhattacharya S, Chatterjee A, Ghosh MK. Current Understanding on EGFR and Wnt/beta-Catenin Signaling in Glioma and Their Possible Crosstalk. Genes Cancer. 2013;4(11-12):427-46.
60. Marikawa Y, Elinson RP. beta-TrCP is a negative regulator of Wnt/beta-catenin signaling pathway and dorsal axis formation in Xenopus embryos. Mech Dev. 1998;77(1):75-80.
61. Liu C, Kato Y, Zhang Z, Do VM, Yankner BA, He X. beta-Trcp couples beta-catenin phosphorylation-degradation and regulates Xenopus axis formation. Proc Natl Acad Sci U S A. 1999;96(11):6273-8.
62. Clevers H, Nusse R. Wnt/beta-catenin signaling and disease. Cell. 2012;149(6):1192-205.
63. Chitalia V, Shivanna S, Martorell J, Meyer R, Edelman E, Rahimi N. c-Cbl, a ubiquitin E3 ligase that targets active beta-catenin: a novel layer of Wnt signaling regulation. J Biol Chem. 2013;288(32):23505-17.
64. Xue J, Chen Y, Wu Y, Wang Z, Zhou A, Zhang S, et al. Tumour suppressor TRIM33 targets nuclear beta-catenin degradation. Nat Commun. 2015;6:6156.
65. Brennan CW, Verhaak RG, McKenna A, Campos B, Noushmehr H, Salama SR, et al. The somatic genomic landscape of glioblastoma. Cell. 2013;155(2):462-77.
66. Yada M, Hatakeyama S, Kamura T, Nishiyama M, Tsunematsu R, Imaki H, et al. Phosphorylation-dependent degradation of c-Myc is mediated by the F-box protein Fbw7. EMBO J. 2004;23(10):2116-25.
67. Bredel M, Bredel C, Juric D, Harsh GR, Vogel H, Recht LD, et al. Functional network analysis reveals extended gliomagenesis pathway maps and three novel MYC-interacting genes in human gliomas. Cancer Res. 2005;65(19):8679-89.
68. Hagedorn M, Delugin M, Abraldes I, Allain N, Belaud-Rotureau MA, Turmo M, et al. FBXW7/hCDC4 controls glioma cell proliferation in vitro and is a prognostic marker for survival in glioblastoma patients. Cell Div. 2007;2:9.
69. Zhang G, Zhu Q, Fu G, Hou J, Hu X, Cao J, et al. TRIP13 promotes the cell proliferation, migration and invasion of glioblastoma through the FBXW7/c-MYC axis. Br J Cancer. 2019;121(12):1069-78.
70. Yang Y, Gao X, Zhang M, Yan S, Sun C, Xiao F, et al. Novel Role of FBXW7 Circular RNA in Repressing Glioma Tumorigenesis. J Natl Cancer Inst. 2018;110(3).
71. Yeh CH, Bellon M, Nicot C. FBXW7: a critical tumor suppressor of human cancers. Mol Cancer. 2018;17(1):115.
